# Skin bridging secondary to ingrown toenail

**DOI:** 10.12669/pjms.306.5790

**Published:** 2014

**Authors:** Mehmet Dadaci, Bilsev Ince, Zeynep Altuntas, Haldun Onuralp Kamburoglu, Ozan Bitik

**Affiliations:** 1 Mehmet Dadaci, MD, Department of Plastic, Reconstructive and Aesthetic Surgery, Meram Faculty of Medicine, Necmettin Erbakan University, Konya, Turkey.; 2 Bilsev Ince, MD, Department of Plastic, Reconstructive and Aesthetic Surgery, Meram Faculty of Medicine, Necmettin Erbakan University, Konya, Turkey.; 3 Zeynep Altuntas, MD, Department of Plastic, Reconstructive and Aesthetic Surgery, Meram Faculty of Medicine, Necmettin Erbakan University, Konya, Turkey.; 4 Haldun Onuralp Kamburoglu, MD, Department of Plastic, Reconstructive and Aesthetic Surgery, Faculty of Medicine, Hacettepe University, Ankara, Turkey.; 5 Ozan Bitik, MD, Department of Plastic, Reconstructive and Aesthetic Surgery, Faculty of Medicine, Hacettepe University, Ankara, Turkey.

**Keywords:** Ingrown toenail, Frost classification, Heifetz classification, Skin bridging

## Abstract

Ingrown toenails are painful conditions that especially affect young people and may become chronic if not treated. We describe a case of chronically inflamed ingrown toenail left untreated for three years. In the physical examination, skin bridging and epithelialization was observed in midline secondary to soft tissue hypertrophy of the lateral nail matrixes. Epithelized fibrous tissue was cut across the lateral nail matrix and left for secondary healing. Partial matrixectomy was applied and the remnants were cauterized in compliance with the Winograd procedure after removal of the nail. Our case is an advanced condition which is the second report in the literature. Skin bridging secondary to excess soft tissue hypertrophy can be observed in untreated bilateral Heinfert or Frost stage 3 ingrown nails. This rare case can be classified as advanced stage 3 disease or stage 4.

## INTRODUCTION

Ingrown toenails are painful conditions that especially affect young people and may become chronic if not treated. Most common causes of ingrown toenails are incorrect clipping of nails, wearing tight shoes, obesity, trauma to toes and/or nails, hyperhidrosis, fungal infection, and differential growth of nails and toes during puberty. The most frequent symptoms are pain, swelling, redness, and suppuration.^1^^-^^4^

Ingrown toenails are classified in three stages as defined by Heifetz^1^ and Frost^2^^,^^3^ (Table-I). Conservative treatment is favored in mild cases, while surgery is necessary for stages 2 and 3 ingrown toenails. Accurate cutting of nails, avoiding tight shoes, application of gauze or special plastic material between the ingrown toenail and flesh, and chiropody are considered as conservative treatment to prevent infection. Surgical treatment is based on partial or total removal of ingrown nail, followed by surgical or chemical destruction of lateral matrix to prevent nail reforming in that area.^3^^-^^7^

In our report we describe a case of chronically inflamed ingrown nail left untreated for three years in which skin bridging and epithelialization occurred in midline secondary to soft tissue hypertrophy of the lateral nail matrixes.

## CASE HISTORY

**Fig.1:**
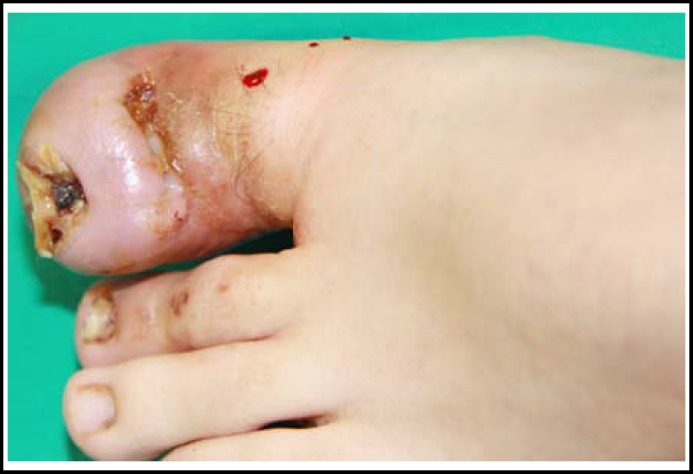
Preoperative photograph.

**Fig.2:**
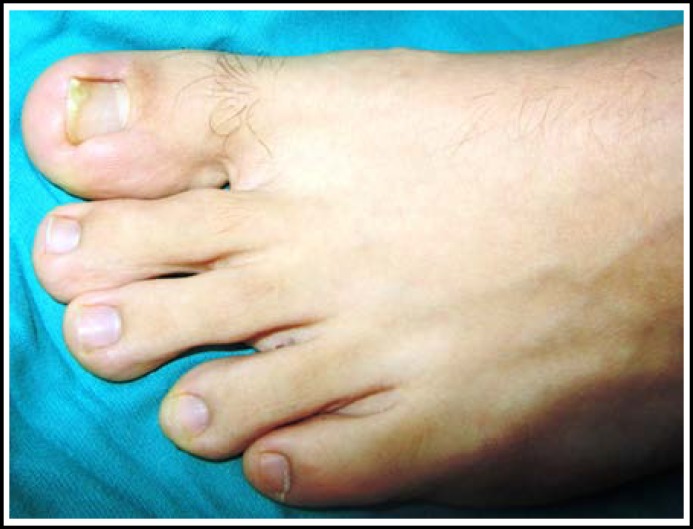
18 months after operation.

A 17-year-old boy was referred to our clinic with the complaint of ingrown nail in his left toe. The patient reported that his complaints started three years ago, but he neither told his family nor referred to any health center because he was afraid of injections. In his physical examination, hypertrophic granulation tissue originating from the lateral nail matrixes was found to be bridged and epithelized in the midline, leaving the lunula and the distal part of the nail unaffected. The toe was edematous and chronically inflamed (Fig.1). We have learned that the granulation tissues have bridged for a year from the patient’s history. Taking the psychological status of the patient into consideration, surgical procedure was conducted with digital block anesthesia and finger tourniquet under sedation. After cleaning the area with povidone-iodine, the granulation tissue was cut in the midline and elevated to explore the nail. The central part of the nail was normal but there was granulation tissue in both lateral matrixes which was epithelized and fibrotic secondary to chronic inflammation. Epithelized fibrous tissue was cut across the lateral nail matrix and left for secondary healing. The nail was removed. Partial matrixectomy was applied and the remnants were cauterized in compliance with the Winograd procedure. Then, the skin on the lateral side was closed with 3-0 prolene suture. After completion of the surgery, dressing was applied with topical antibiotics. Dressing with Sodium Fusidat pomade was changed in every two days. Postoperatively, amoxycillin-clavulanic acid (1gr) and non-steroidal anti-inflammatory drugs were prescribed twice daily. Sutures were removed on the postoperative 20^th^ day. The appearance of the patient’s finger on the postoperative 18^th^ month is shown in Fig.2.

## DISCUSSION

This case report demonstrated that if ingrown nails are bilateral and left untreated for a long time, hypertrophic granulation tissue can transform into epithelized fibrous tissue and bridge in the midline over the nail. Our case is the second report in the literature. 

**Table-I:** Heifetz and Forest classifications for ingrown toenails.

**Heifetz classification for ingrown toenails**
**Stage 1** Slight erythema and swelling of the nail grooves in the nail bed.
**Stage 2** Presence of acute infection and suppuration.
**Stage 3** Chronic infection, the formation of granulation tissue surrounding the nail groove and hypertrophy of the surrounding tissues.
**Forest classification for ingrown toenails**
**Stage 1 **Nails have spur formation in the lateral nail fold that occurs due to irregular nail growth in a normal nail bed.
**Stage 2** Nails have inwards folding of the lateral border of the nail bed (concave nail).
**Stage 3** Nails have a normal nail bed accompanied by soft tissue hypertrophy in the lateral border, hypertrophy of the surrounding tissues.

In Heinfetz and Frost classification the last stage is Stage 3. This stage is described as the presence of chronic infection, formation of granulation tissue surrounding the nail groove and hypertrophy of the surrounding tissues. Our case is an advanced condition which is not present in this classification.^1^^-^^4^ Seyfettinoglu et al.^8^ reported a similar case of skin bridging secondary to bilateral ingrown nails. They reported that the nail was obscured by extreme soft tissue hypertrophy but our case is more advanced. The differences of our case are the younger age of the patient, long duration of the disease, presence of chronic infection, excessive edema of the toe and the higher degree of soft tissue hypertrophy. Both cases do not enter the classifications described up to now.

Our case hided his disease and did not refer to hospital for 3 years because of his injection phobia. Needle phobia affects an estimated 4% of the general population. Phobia attacks usually manifest with sympathetic over activity in the form of tachycardia, hypertension, headache, agitation, and hyperventilation. Phobia attacks may also present with parasympathetic activity. Especially needle phobia are commonly associated with a vasovagal response, which is an inappropriate combination of vasodilation and bradycardia resulting in collapse, syncope and rarely in asystole. In these patients preoperative phobia desensitization therapy should be applied if possible. Alternatively, benzodiazepine sedation or EMLA can be suggested.^9^^,^^10^ In our case, surgery was completed without complications under sedation and local anesthesia. Although local anesthesia is sufficient for this procedure, sedation is needed for patients with a high level of anxiety.

For conservative management of ingrown nails; accurate cutting of nails, avoiding tight shoes, application of gauze or special plastic material between the ingrown nail and flesh, chiropody, and treatment of potential infections are recommended. In distal nail ingrowths, cure can be provided with conservative treatment in stage 1. Surgery is inevitable otherwise. Surgery depends on surgical or chemical destruction of the lateral matrix after total or partial removal of the nail. One year recurrence rates were reported between 5.5% to 40% in different techniques.^3^^-^^7^ In this rare case, we removed the bridging granulation tissue up to the lateral nail matrix and left for secondary healing.

Skin bridging secondary to excess soft tissue hypertrophy can be observed in untreated bilateral Heinfert or Frost stage 3 ingrown nails. This rare case can be classified as advanced stage 3 disease or stage 4 disease.

## Authors; Contribution:

**MD and BI** performed the surgery and followed the patient. **MD and ZA **did data collection and manuscript writing. **MD, HOK and OB **did review and final approval of manuscript.

**MD** takes the responsibility and is accountable for all aspects of the work in ensuring that questions related to the accuracy or integrity of any part of the work are appropriately investigated and resolved.

## Notes

***Funding statement:*** The research was not sponsored by an outside organization. This study conformed to the Helsinki Declaration.
